# Factors predicting good prognosis of failed intra-arterial thrombectomy cases: A retrospective study

**DOI:** 10.1097/MD.0000000000033866

**Published:** 2023-05-26

**Authors:** Hyunjun Jo, In-Hyoung Lee, Sung-Kon Ha, Dong-Jun Lim, Jong-Il Choi

**Affiliations:** a Department of Neurosurgery, Korea University Guro Hospital, Korea University College of Medicine, Seoul, Korea; b Department of Neurosurgery, Korea University Ansan Hospital, Korea University College of Medicine, Ansan-si, Korea.

**Keywords:** acute ischemic stroke, intra-arterial thrombectomy, leptomeningeal collateral flow, susceptibility-weighted imaging

## Abstract

Intra-arterial thrombectomy (IAT) has been increasingly applied in the treatment of acute ischemic stroke (AIS) due to large-vessel occlusion, and many related studies have been published. However, limited studies on the prognosis of failed-IAT patients are available. In this study, factors that can predict a good prognosis in patients with failed IAT were studied. Among patients who visited our hospital between January 2016 and September 2022 and underwent IAT, we retrospectively analyzed those with failed IAT. A univariate analysis was performed on the radiological features, medical histories, and other patient characteristics expected to affect the prognosis, and a multivariate analysis was performed on some of these factors. In univariate analysis, a good collateral channel on susceptibility-weighted imaging (SWI), modified thrombolysis in cerebral infarction (mTICI) 2A recanalization, and the pre-procedural modified Rankin scale (mRS) were statistically significant. In the multivariate analysis, good collateral channels on SWI and computed tomography angiography (CTA) and mTICI 2A recanalization were statistically significant. Factors that can predict a good prognosis in patients with failed IAT include good leptomeningeal collateral channels evaluated by CTA and SWI and mTICI 2A recanalization.

## 1. Introduction

In recent years, the treatment of acute ischemic stroke (AIS) has evolved.^[1–7]^ In particular, the treatment of AIS with large-vessel occlusion is rapidly evolving with the introduction of intra-arterial procedures.^[1–8]^ The indications are also being expanded through several large-scale studies, and the time limit is gradually increasing.^[1–3]^ Even a recent American Heart Association/American Stroke Association (AHA/ASA) study reported that as perfusion studies such as computed tomography perfusion or magnetic resonance imaging perfusion develop, clinicians should decide whether to perform reperfusion therapy by not just relying on the time window but determining it with perfusion imaging.^[3]^

However, despite the development of instruments such as upgraded guiding catheters, microcatheters, and stent retrievers, the success rate of intra-arterial thrombectomy (IAT) is reported to be approximately 80%, which means that a failure rate of approximately 20% still exists.^[7,9–14]^ The prognosis of IAS in a patient differs significantly depending on whether IAT is successful. However, various factors can affect the prognosis in a patient, such as the time from onset to the success of IAT or the patient leptomeningeal collateral channel.^[15–18]^ Furthermore, because various factors affect the patient prognosis, even if the IAT fails, the prognosis is not necessarily poor.^[19]^ Even if the IAT fails, if the prognosis of the case can be predicted in advance, it will help decide whether to apply rescue treatment, such as intracranial stent insertion or extracranial-to-intracranial (EC-IC) bypass. However, fewer studies on the factors that can predict a good prognosis for patients with IAT failure are available. In this study, we investigated the factors that can predict good outcomes in patients with failed IAT.

## 2. Methods

### 2.1. Study design and procedure indications

This retrospective study was approved by the Institutional Review Board of the Korea University Ansan Hospital (2022AS0103). We retrospectively examined patients who visited our hospital between January 2016 and September 2022 and underwent IAT. Patients treated for posterior circulation lesions and cases treated for in-stent thrombosis after stent-assisted coil embolization or flow-diverting stent insertion were excluded. Patients who underwent rescue treatment, such as an EC-IC bypass, after IAT failure were also excluded.

We performed IAT based on 6 positive “early window” mechanical thrombectomy trials,^[20–25]^ which were based on the 2018 AHA/ASA Early Stroke Management Guidelines^[26]^; that was a patient with AIS with occlusion of the internal carotid artery (ICA) or proximal middle cerebral artery having a National Institutes of Health stroke scale score ≥6 and Alberta Stroke Program Early Computed Tomography (ASPECT) score ≥6 within 6 hours of the last known normal or within 6 to 24 hours of the last known normal as well as having a ratio of the volume of ischemic tissue on the perfusion study to the infarction volume of 1.8 or more.^[26]^ Tissue plasminogen activator was administered in patients who were non-contraindicated for ITA according to the judgment of the neurologist before IAT. The time from the patient last normal time to the femoral artery puncture and the time from the first abnormal time to the puncture were recorded.

Patients with AIS who visited our hospital underwent brain computed tomography (CT), diffusion magnetic resonance imaging (MRI), or both, according to the judgment of the neurologist in the emergency room. If CT angiography (CTA) or magnetic resonance angiography was performed if deemed necessary. Among the patients, those who underwent examination using both CTA and MRI-susceptibility-weighted imaging (SWI) were included to compare the efficacy of CT and MRI in the judgment of leptomeningeal collateral channels without patient selection bias.

### 2.2. Interventional procedure

The right femoral artery was punctured under local anesthesia. 8Fr long sheath was inserted, and the proximal ICA was selected with an 8Fr balloon guiding catheter. The lesion site was approached using a 5Fr aspiration catheter. After passing through the lesion site with a microcatheter, a stent retriever was deployed over the lesion site. The procedure was repeated up to 3 times; however, if the thrombectomy failed despite 3 attempts, it was terminated to avoid complications such as intracerebral hemorrhage (ICH). We attempted to check the number of thrombectomy attempts; however, some of the old records were missing. Therefore, we evaluated the total procedure time indirectly.

### 2.3. Radiological analysis

#### 1.2.3. CT-based assessment.

**Figure 1.**
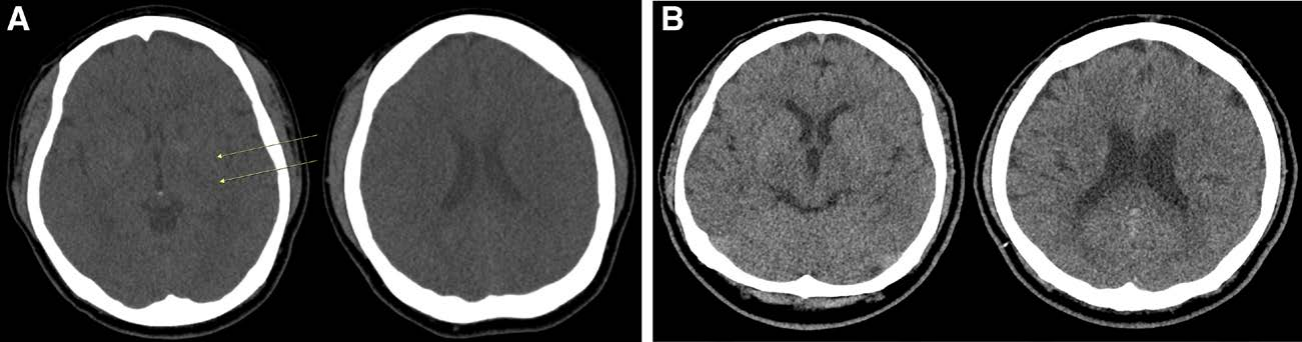
Examples of the Alberta Stroke Program Early Computed Tomography (ASPECT) score evaluation. (A) an example of a patient with a poor ASPECT score of 7, showing slightly decreased intensity in the left putamen, internal capsule, and insular cortex. (B) an example of a patient with a good ASPECT score of 10, showing no decreased intensity.

**Figure 2.**
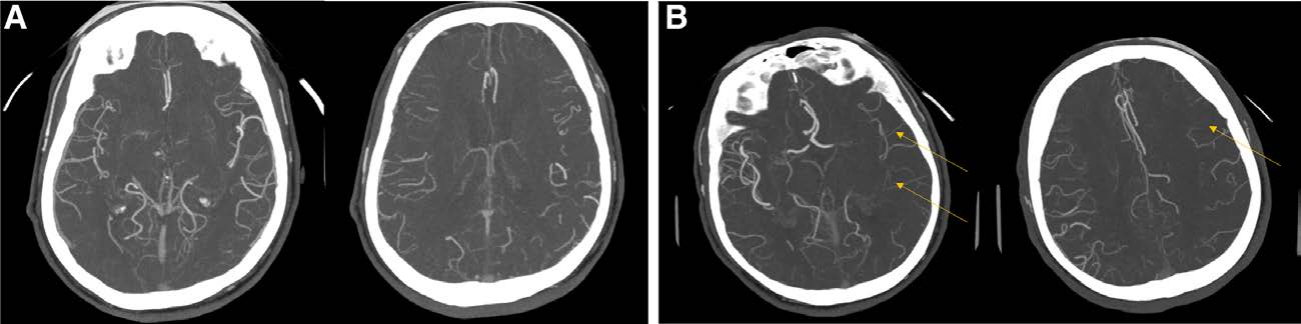
According to the study of Menon et al, examples of evaluation of collateral flow with computed tomography angiography (CTA). (A) a patient who was diagnosed with right M1 occlusion and underwent IAT, which was evaluated at 15 points and judged to be a good collateral channel. (B) a patient diagnosed with left distal ICA occlusion, which was evaluated as 9 points and judged to have a poor collateral channel. IAT = intra-arterial thrombectomy, ICA = internal carotid artery.

First, the ASPECT^[27]^ score was evaluated to determine its association with the prognosis. An ASPECT score of 8 or higher was classified as good. In addition, the presence or absence of a dense middle cerebral artery sign, which is known to be indicative of an embolus, was assessed. Furthermore, if there was calcification of the site of the intracranial artery on brain CT, the possibility of in situ thrombosis was assumed to be high, and this was also evaluated. Based on previous studies, we evaluated the patient leptomeningeal collateral channel using raw CTA data.^[28]^ Six ASPECT regions (M1-6), the anterior cerebral artery, and the basal ganglia were evaluated in comparison with the normal contralateral hemisphere (0, no; 1, less; 2, equal). The Sylvian sulcus was evaluated at 0, 2, and 4 points in the same way. A total score of 11 or higher was judged as a good collateral channel, and a score of 10 or lower was judged as a poor collateral channel. To simplify the existing evaluation method, the existing “poor” group corresponding to 0 to 10 points was evaluated as “poor,” and the existing “medium” corresponding to 11 to 16 points, and “good” corresponding to 17 to 20 points were combined into one, being evaluated as “good.” Finally, a postoperative CT was performed to confirm the occurrence of ICH. Examples of CT-based evaluation of the ASPECT score and leptomeningeal collateral channel are shown in Figures 1 and 2, respectively.

#### 2.2.3. MRI-based assessment.

**Figure 3.**
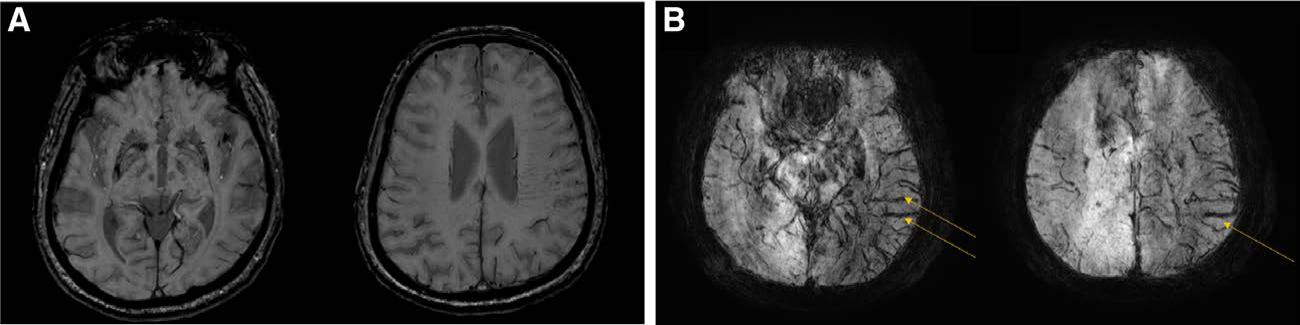
Examples of collateral flow evaluation with susceptibility-weighted imaging (SWI), according to the study of Lee et al (A) SWI of the patient as in Figure 2A, and it was judged to be a good collateral channel consistent with computed tomography angiography (CTA). (B) SWI images of the patient as in Figure 2B, and it was judged to be a poor collateral channel, which is the same as in CTA. In this manuscript, 2 cases were shown to present a representative image, so the collateral channel judged by CTA and SWI was the same. However, in some cases, the collateral channel judged by CTA and SWI did not match.

To evaluate the leptomeningeal collateral channel, SWI was used for grading according to existing studies.^[29,30]^ A good collateral channel was judged to exist when there was no prominent cortical or medullary vein in the affected hemisphere or only mildly prominent cortical and/or medullary veins. Moderate or very prominent cortical or medullary veins in the affected hemisphere were judged to be poor collateral channels. We also simplified the preexisting SWI-based collateral evaluation methods. Patients rated as “poor” and “very poor” were evaluated as “poor,” and those rated as “intermediate” and “good” were evaluated as “good.” Examples of MRI-based evaluations of leptomeningeal collateral channels are shown in Figure 3.

#### 3.2.3. TFCA-based assessment.

**Figure 4.**
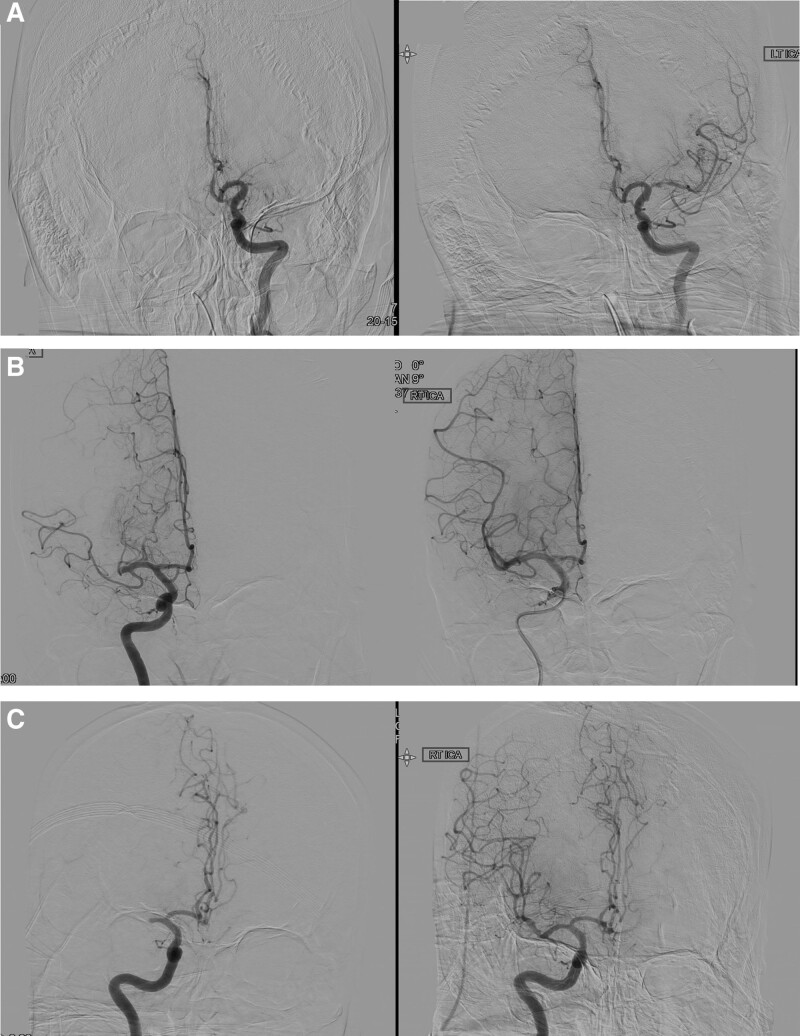
Examples of judging the recanalization grade according to the modified thrombolysis in cerebral infarction (mTICI) scoring system. (A) Grade 2A recanalization, in which less than half of the occluded territory is refilled. (B) Recanalization of more than half of the occluded flow, which means grade 2B recanalization. (C) Grade 2C recanalization, which shows near complete reperfusion with a slow flow.

In situ thrombosis was determined through transfemoral cerebral angiography (TFCA) performed during IAT. Using TFCA, we analyzed whether any factors can predict in situ thrombosis in imaging studies performed before the intervention. The degree of recanalization was evaluated using the modified thrombolysis in cerebral infarction (mTICI) grading system^[6,31,32]^ and grade 2B or better was judged successful recanalization. Among the failed IATs, 2A recanalization was also evaluated separately to confirm whether there was a difference in prognosis between 2A recanalization and 1 recanalization. Examples of TFCA-based evaluations using the mTICI grading system are shown in Figure 4.

### 2.4. Clinical outcomes

To analyze the patients’ clinical results, the preoperative modified Rankin Scale (mRS) was used, and the results were compared with the mRS score at the last follow-up. Postoperatively, an mRS score of ≤2 was defined as a good outcome, and ≥3 was defined as a bad outcome.

### 2.5. Statistical analysis

Statistical analyses were performed using IBM SPSS Statistics version 25 (SPSS, Chicago, Illinois). Continuous variables are presented as medians (interquartile range). Univariate logistic regression was performed to confirm whether there were factors that could predict good outcomes after IAT, following which a multivariable analysis was performed only for the variables with *P* values lower than .1 in the univariate analysis. Statistical significance was set at a *P* value <.05 in this study.

## 3. Results

### 3.1. Baseline characteristics

**Figure 5.**
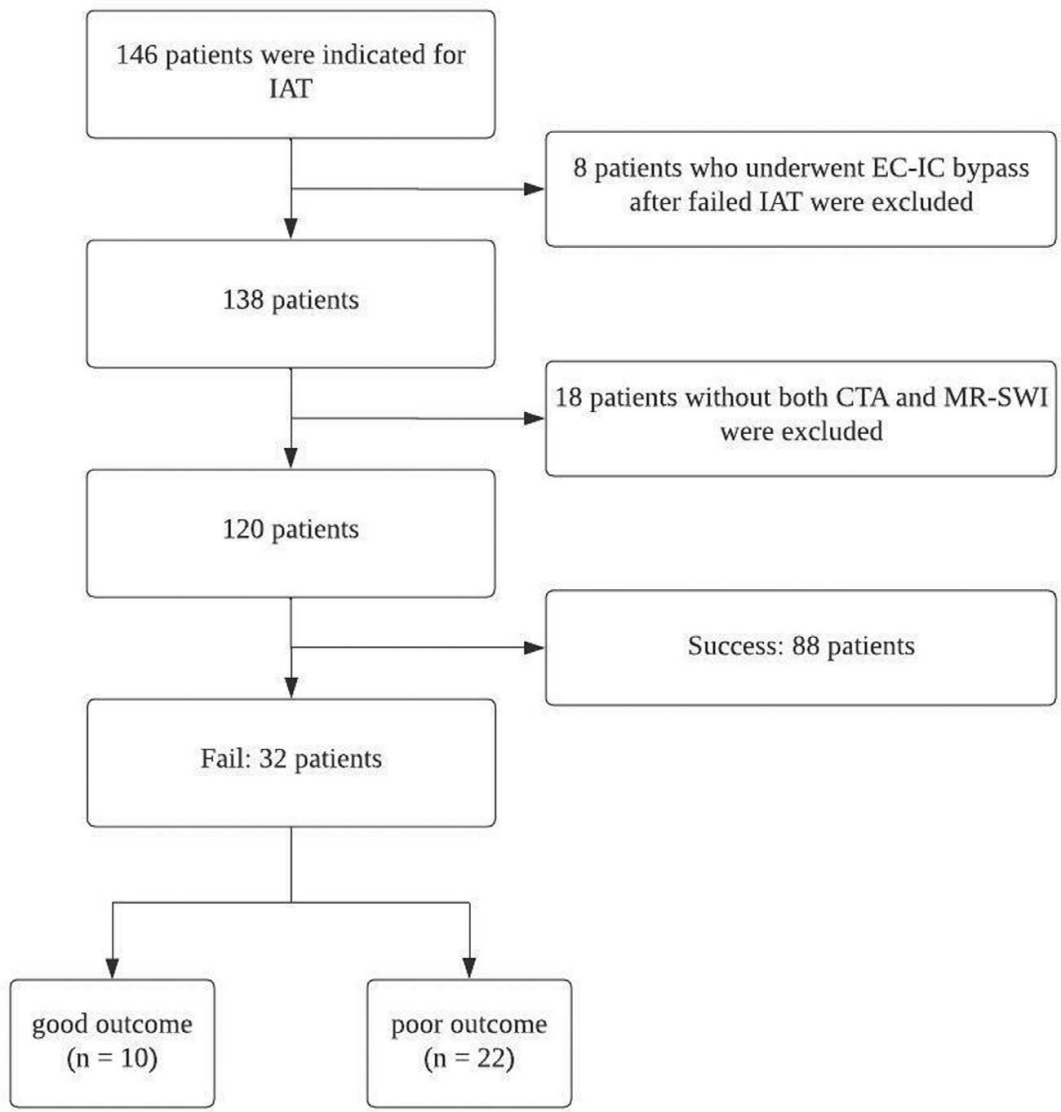
Flow diagram of the enrollment process of the patients.

**Figure 6.**
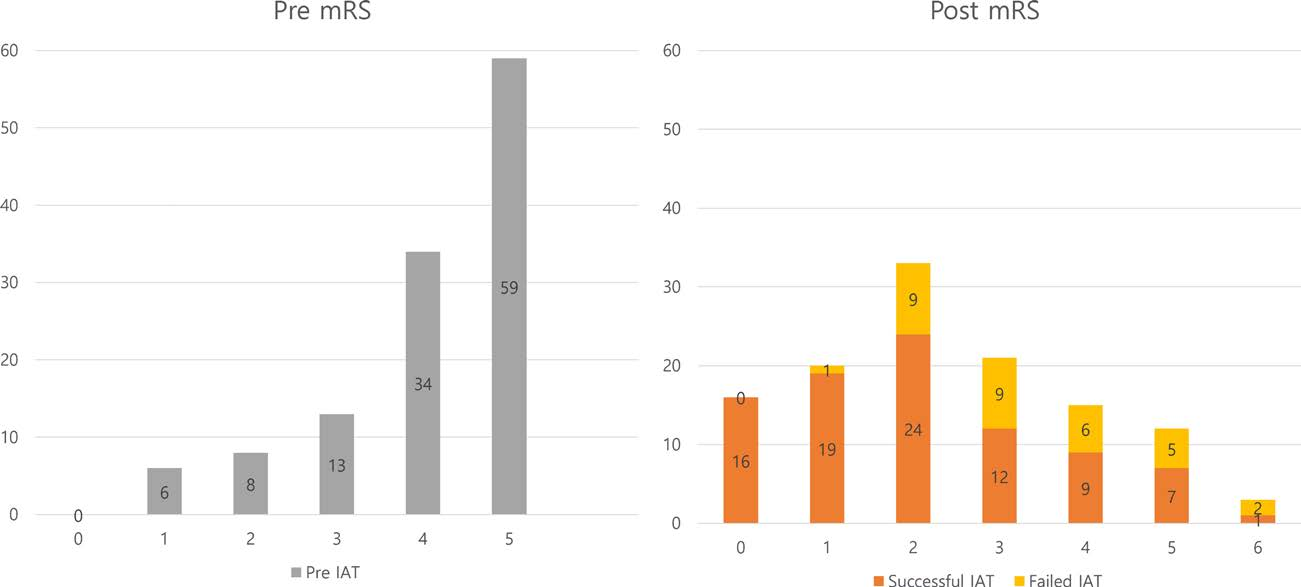
Comparison of pre- and post-intra-arterial thrombectomy (IAT) modified Rankin Scale (mRS). Before the IAT, only 2.2% (2/90) of the patients had an mRS score of 2 or better, but after the IAT, 58.9% (53/90) of the patients had an mRS score of 2 or better. Among them, 67.1% (47/70) of patients with successful IAT show a good outcome, and 30% (6/20) of failed IAT patients show a good outcome.

We enrolled 32 patients after excluding patients based on the exclusion criteria mentioned above (Fig. 5). The median age of the patients was 64.0 (46.25–75.5) years, and 16 (50.0%) patients were male (Table 1). Based on the last normal time to puncture time, it took 227.0 (210.0–349.5) minutes, and based on the first abnormal time to puncture time, it took 227 (210.0–310.75) minutes. The underlying diseases of the patients are listed in Table 1. The procedure took 80.5 (47.0–101.75) minutes. According to national guidelines, tissue plasminogen activator was administered to 22 (68.8%) of the patients.^[26]^ The preprocedural mRS, along with the postprocedural mRS, are shown in Figure 6, comparing successful and failed IAT cases. No statistically significant differences in the baseline characteristics were observed between patients with good outcomes and those with poor outcomes.

**Table 1:** Patients’ baseline characteristics.

Variables	Total	Good outcome	*P* value
		Bad outcome	
Age*	64.0 (46.25–75.5)	61.0 (36.0–67.0)	.251
		64.0 (54.5–77.0)	
Sex (male (%))	16 (50.0)	5 (50.0)	1.000
		11 (50.0)	
Side (right (%))	17 (53.1)	4 (40.0)	.316
		13 (59.1)	
Follow-up period*	43.0 (35.0–49.75)	44.5 (36.75–50.0)	.163
		38.0 (29.75–48.5)	
Location of lesion (%)			
Distal ICA	14 (46.9)	5 (50.0)	.631
		9 (40.9)	
M1	18 (53.1)	5 (50.0)	
		13 (59.1)	
Medical history, number (%)			
Hypertension	16 (50.0)	4 (40.0)	.446
		12 (54.5)	
Diabetes mellitus	6 (18.8)	2 (20.0)	.903
		4 (18.2)	
Hyperlipidemia	3 (9.4)	1 (10.0)	.935
		2 (9.1)	
Atrial fibrillation	6 (18.8)	2 (20.0)	.903
		4 (18.2)	
Last normal time to puncture time*	227.0 (210.0–349.5)	266.5 (210.0–350.0)	.617
		227.0 (180.0–310.5)	
First abnormal time to puncture time*	227.0 (210.0–310.75)	232.5 (210.0–350.0)	.617
		227.0 (180.0–292.0)	
Procedure time*	80.5 (47.0–101.75)	83.5 (56.75–122.75)	.434
		75.5 (45.75–97.25)	
Use of tPA	22 (68.8)	8 (80.0)	.355
		12 (54.5)	

ICA = internal carotid artery, tPA = tissue plasminogen activator.

* Median (interquartile range).

### 3.2. Factors that can predict the good prognosis of the failed IAT patients

We conducted a univariate analysis to analyze the factors predicting good outcomes among patients with failed IAT. The results of the univariate analysis showed that a good leptomeningeal collateral channel on SWI (odds ratio 4.167, *P* = .034), mTICI 2A recanalization (odds ratio 3.125, *P* = .042), and pre-procedural mRS (odds ratio 0.085, *P* = .039) were statistically significant factors. Multivariate analysis was performed by including factors with a *P* value ≤.1 in univariate analysis. In multivariate analysis, a good leptomeningeal collateral channel on CTA (odds ratio 9.500, *P* = .012) and SWI (odds ratio 11.989, *P* = .017) and mTICI 2A recanalization (odds ratio 3.505, *P* = .050) were significant factors (Table 2).

**Table 2:** Univariate and multivariate analysis of factors affecting good outcome in patients with failed intra-arterial thrombectomy.

Univariate analysis			
Variables	Odds ratio	95% confidence interval	*P* value
Age	0.974	0.911–1.040	.430
Sex			
Male	Reference	–	–
Female	1.000	0.148–6.772	1.000
Side			
Right	Reference	–	–
Left	0.500	0.068–3.675	.496
Location of lesion			
Distal ICA	Reference	–	–
M1	0.556	0.080–3.858	.552
Hypertension	1.500	0.203–11.088	.691
Diabetes mellitus	0.000	0.000–	1.000
Hyperlipidemia	0.000	0.000–	1.000
Atrial fibrillation	3.000	0.312–28.841	.341
Last normal time to puncture time	1.000	0.995–1.005	.912
First abnormal time to puncture time	0.996	0.982–1.010	.558
Puncture to recanalization time	1.023	0.994–1.052	.117
Use of tPA	1.500	0.203–11.088	.691
Good ASPECT	0.904	0.306–2.671	.855
Calcification	0.375	0.051–2.772	.337
Dense MCA sign	5.000	0.640–39.059	.125
CTA collateral			
Poor	Reference	–	–
Good	4.083	0.818–20.376	.086
SWI collateral			
Poor	Reference	–	–
Good	4.167	0.363–47.811	.034
In Situ thrombosis	0.556	0.080–3.858	.552
mTICI 2A recanalization	3.125	0.272–35.858	.042
Pre mRS	0.085	0.008–0.878	.039
Pre NIHSS	0.310	0.718–1.111	.310
Multivariate analysis			
Variables	Odds ratio	95% confidence interval	*P* value
CTA collateral			
Poor	Reference	–	–
Good	9.500	1.641–54.994	.012
SWI collateral			
Poor	Reference	–	–
Good	11.989	1.900–75.670	.017
mTICI 2A recanalization	3.505	1.884–5.556	.050
Pre mRS	0.014	0.000–1.355	.167

ASPECT = Alberta Stroke Program Early CT, ASPEDM = Alberta Stroke Program Early diffusion MR, CTA = computed tomography angiography, ICA = internal carotid artery, ICH = intracerebral hemorrhage, MCA = middle cerebral artery, MI = myocardial infarction, mRS = modified Rankin Scale, NIHSS = National Institutes of Health Stroke Scale, SWI = susceptibility-weighted imaging, tPA = tissue plasminogen activator.

## 4. Discussion

IAT is an important part of the recent treatment options available for AIS. Our treatment results over the past 6 years have shown similar success rates (88/120, 73.3%) to those in other studies, and the success rate has gradually increased over time compared with those in the past. The success rate in the last 3 years was 79.1% (53/67), which was higher than the previously reported success rate of 66.0% (35/53) and is thought to be owing to the development of instruments such as catheters and stent retrievers and the methodological establishment of the IAT.

As IAT gradually develops, its indications are gradually expanding.^[1–3]^ In the past, only the time from the onset of symptoms was the standard, but perfusion-diffusion mismatch or perfusion-symptom mismatch gradually became the standard for indications.^[3,26]^ An increasing number of patients can benefit from IAT by conducting individualized assessments through various evaluation methods instead of evaluating patients based on a uniform standard of time from onset. This is probably because of the difference in the collateral flow system for each patient. Several studies have reported that the leptomeningeal collateral channel is an important factor in the prognosis of patients; in particular, it was observed to be an important factor in our study on patients with failed IAT.

Regarding the modality of evaluating the collateral flow system, there are methods using CT and MRI, both of which are useful in evaluating the collateral channels. However, the evaluation method using CT is less convenient than the method using MRI because it is a method of summing scores from several parts, similar to evaluating ASPECT scores. In contrast, the SWI method evaluates the collateral channel by judging the presence of prominent cortical or medullary veins. Previous studies have claimed that the appearance of prominent cortical and medullary veins is caused by an increase in the ratio of deoxyhemoglobin to oxyhemoglobin due to a mismatch between oxygen supply and demand in hypoperfused tissue.^[29,30]^ In other words, if the presence of prominent cortical or medullary veins is confirmed in SWI, the collateral channel of that part is believed to be not good.^[29,30]^ Compared with the method using CT, the evaluation method using SWI is more convenient because it does not evaluate the collateral channel in each region but instead evaluates the presence of a prominent vein. Nevertheless, it is considered a significant factor in predicting prognosis, as in the collateral channel evaluation method using CTA. With the development of MRI, SWI has become a widely used imaging method. Therefore, if SWI is performed along with diffusion MRI without the need to deliberately perform CTA to evaluate collateral channels, it is expected that collateral channels can be properly evaluated without the use of a contrast agent.

Since the TICI grading system was first published, there have been 2 modifications, and several studies have shown that the prognosis of the patient is better when mTICI 2C or 3 is achieved rather than 2B, which is the general standard for successful recanalization.^[33,34]^ However, few studies have shown that 2A recanalization is more beneficial to patient prognosis than recanalization of 1 or less, even in cases of failed IATs of 2A or less.^[16]^ Through our study, we can assert that failure at a higher grade has a meaningful impact on patient prognosis. The first possible reason may be that even if recanalization is achieved only below 50%, it can send a small amount of flow to the ischemic penumbra, and compared to that, it hardly recovers flow similar to that in mTICI grade 1. The second possible reason may be that even if only 2A recanalization is achieved, the flow of the lenticulostriate artery originating from M1 and supplying deep cortical structures, such as the basal ganglia and internal capsule, can be partially restored. To analyze this more accurately, it is necessary to analyze whether the original lesion was the distal ICA and if it was M1, whether the lenticulostriate artery was occluded or not. A larger-scale study is needed in the future.

In summary, good prognostic factors for AIS patients with failed IAT were good collateral for CT or MR SWI and mTICI 2A recanalization. Our study results can provide a good reference for selecting various treatment options when performing IAT. When the IAT fails and the prognosis is expected to be poor, rescue treatments such as rescue stent insertion, or an EC-IC bypass may be considered. However, even if the IAT is unsuccessful, if the patient prognosis is not considered to be bad, clinicians may choose to stop the procedure to avoid unexpected complications such as ICH while still forcing the IAT.

Our study has the limitation of being retrospective. In addition, there is a disadvantage in that the number of patients enrolled in the study was relatively small, despite the exclusion criteria. A better-designed, large-scale study in the future is needed.

## 5. Conclusion

Factors that can predict a good prognosis in patients with failed IAT include a good leptomeningeal collateral channel evaluated by CTA and SWI and mTICI 2A recanalization.

## Acknowledgments

Statistical analysis was consulted to our hospital statistical counseling center.

## Author contributions

**Conceptualization:** Sung-Kon Ha, Dong-Jun Lim, Jong-Il Choi.

**Data curation:** Hyunjun Jo, In Hyoung Lee, Sung-Kon Ha, Dong-Jun Lim.

**Formal analysis:** Hyunjun Jo, In Hyoung Lee.

**Funding acquisition:** Jong-Il Choi.

**Investigation:** Hyunjun Jo, In Hyoung Lee, Jong-Il Choi.

**Methodology:** Sung-Kon Ha, Jong-Il Choi.

**Project administration:** Jong-Il Choi.

**Resources:** Jong-Il Choi.

**Software:** Hyunjun Jo.

**Supervision:** Jong-Il Choi.

**Validation:** Hyunjun Jo, Jong-Il Choi.

**Writing – original draft:** Hyunjun Jo.

**Writing – review & editing:** Hyunjun Jo, Jong-Il Choi.
